# ccTSA: A Coverage-Centric Threaded Sequence Assembler

**DOI:** 10.1371/journal.pone.0039232

**Published:** 2012-06-19

**Authors:** Jung Ho Ahn

**Affiliations:** Department of Intelligent Convergence Systems, Seoul National University, Seoul, Republic of Korea; University of Maryland, United States of America

## Abstract

De novo sequencing, a process to find the whole genome or the regions of a species without references, requires much higher computational power compared to mapped sequencing with references. The advent and continuous evolution of next-generation sequencing technologies further stress the demands of high-throughput processing of myriads of short DNA fragments. Recently announced sequence assemblers, such as Velvet, SOAPdenovo, and ABySS, all exploit parallelism to meet these computational demands since contemporary computer systems primarily rely on scaling the number of computing cores to improve performance. However, most of them are not tailored to exploit the full potential of these systems, leading to suboptimal performance. In this paper, we present ccTSA, a parallel sequence assembler that utilizes coverage to prune k-mers, find preferred edges, and resolve conflicts in preferred edges between k-mers. We minimize computation dependencies between threads to effectively parallelize k-mer processing. We also judiciously allocate and reuse memory space in order to lower memory usage and further improve sequencing speed. The results of ccTSA are compelling such that it runs several times faster than other assemblers while providing comparable quality values such as N50.

## Introduction

Sequence assembly is a process of aligning and merging the fragments of a DNA sequence to reconstruct the original one, which is an important part of bioinformatics [1]. It can be categorized into two types, mapped and de-novo assembly. The mapped assembly has a reference sequence for the fragments to be assembled, while the de-novo assembly does not. A de-novo assembler is used to find the complete DNA sequence of an organism without a reference genome and to assemble some parts of the sequence that are largely different from the reference. The lack of a reference sequence makes a de-novo assembler demand much higher computational power than a mapped assembler to assemble the same amount of fragments [1], [2].

The advent and continuous evolution of next-generation sequencing (NGS) machines enable the high-throughput sequencing of short DNA fragments called reads [3], [4], whose length is typically in the range of dozens to low hundreds of base pairs. Traditional assembly methods, such as Smith-Waterman algorithm [5], are not suitable to process these massive data effectively. Instead, recently announced sequence assemblers such as Velvet [6], ABySS [7], and SOAPdenovo [8] extract fixed length k-mers from the reads and build de Bruijn graphs using the k-mers. These assemblers [6]–[10] are all parallelized in order to meet the computational demands of de-novo assembly. It is because contemporary computer systems primarily rely on scaling the number of computing cores to improve performance [11]. A system with dozens of cores and terabytes of shared memory was available only as a supercomputer and excessively expensive before, but now it is even cheaper than a sequencing machine.

Even though the parallel versions of these assemblers provide a noticeable improvement in assembly speed, those are not tailored to exploit the full potential of modern computer systems. They either statically divide workload to computing cores or assume message passing between cores. Even if the reads are evenly distributed across the cores, the time taken to build and access a data structure that is storing k-mers heavily depends on the distribution of the k-mer values extracted from the reads as well as the memory system architecture of the computer systems. This can lead to a huge load imbalance problem across the cores [12]. Message passing has been a technique primarily for programs on a cluster of computers connected over a network, where the access latency and communication throughput over the network are an order of magnitude worse than those over shared memory on multithreaded programs [12]. Because a de-novo assembler is a memory intensive application, an application designed for a message passing system typically does not perform effectively on a shared-memory system. These all lead to suboptimal performance.

In this paper, we introduce ccTSA, a coverage-centric threaded sequence assembler, which is written in C++. It utilizes k-mer coverage, the number of k-mer instances in the DNA fragments, in building a k-mer coverage table, pruning k-mers from the table, finding preferred edges in the de Bruijn graph [13] made of k-mer nodes, and resolving conflicts between the preferred edges. It exploits the high-throughput and low-latency memory access characteristics of modern shared-memory systems by spawning multiple worker threads and making them access data structures concurrently in the shared memory. In order to reduce memory usage, ccTSA extensively utilizes bit fields, implements a custom memory allocator [14], and has an option to prune low coverage k-mers in the middle of building the k-mer coverage table, which provides a tradeoff between the memory footprint and assembly quality. The modular structure and careful design make ccTSA run faster and have better scalability in sequencing speed than other sequence assemblers, while providing comparable memory usage and quality values such as N50.

## Results and Discussion

We compared the performance (sequencing speed and memory usage) and quality (such as N50 and NG50) of ccTSA with other sequence assemblers using synthetic reads from 4 organisms and real paired-end reads from 2 organisms. First, synthetic reads were used for comparison, which enabled the results of the assemblers to be compared to the original sequence. The scalability of sequencing speed on ccTSA and other assemblers were evaluated. We utilized the evaluation framework of GAGE [4] to compare the quality of the assemblers using 2 whole-genome shotgun paired-end data. We also explored one of the ccTSA’s interesting features that provides the tradeoff between memory usage and assembly quality by pruning low coverage k-mers in the middle, not at the end, of building a k-mer coverage table.

### Experimental Setup

As for the synthetic reads, we used the datasets of 4 organisms: C.elegans (Caenorhabditis elegans), E.coli (Escherichia coli strain K-12), L.major (Leishmania major strain Friedlin), and S.cerevisiae (Saccharomyces cerevisiae S288c). The reference genome of each organism was downloaded from NCBI Genome Sequence (Table 1, 1, and S2). MetaSim [15] was used to generate synthetic reads for each reference genome. MetaSim provides options to choose a read length, an average sequence coverage value, and an empirical error model. The sequence coverage stands for how many times a nucleotide in the original sequence (the genome of an organism in our study) appears at the reads. We set the read length to either 36 or 75 base pairs (bps), the sequence coverage to 10, 20, 40, 80, or 160, and the empirical error model to either error free (Exact) or an error model for the short reads of the Illumina technology (Illumina). We used the error model included in MetaSim for the error probabilities of 36 bp reads and the one from Plantagora [16] for the probabilities of 75 bp reads. For example, a dataset ‘E.coli-Illumina-**75** bp-80x’ consists of a sequence of reads from the E.coli reference genome with the sequence coverage of 80, each of which has 75 base pairs, and following the Illumina error probability model. All simulation parameters of MetaSim are listed in Table S3. ccTSA relies on separate scaffolding tools to orient and align the contigs into super-contigs or scaffolds. In order to fairly compare the performance and quality of the assemblers, we configured each assembler to treat the synthetic sequences as single-end reads, and excluded scaffolding and gap closure parts from comparison even though MetaSim generated paired-end data.

**Table 1:** Reference genome datasets downloaded from NCBI Genome Sequence.

TaxonomyID	Name	Genomes	Size
6239	Caenorhabditis Elegans	6 (Linear)	100,267,633
31685	Escherichia Coli str. K-12 Substr.DH10B	1 (Circular)	4,686,137
347515	Leishmania Major Strain Friedlin	36 (Linear)	32,816,778
559292	Saccharmoyces Cerevisiae S288c	16 (Linear)	12,071,326

The details of the NGS data we got and used for the experiments are listed in Table S1 and S2.

We used the paired-end whole-genome shotgun data of the following organisms: S.aureus (Staphylococcus aureus) and R.sphaeroides (Rhodobacter sphaeroides). We downloaded the data sets from the GAGE [4] web site at http://gage.cbcb.umd.edu, which originated from NCBI Genome Sequence, and then were preprocessed using the Quake [17] and ALLPATHS-LG [18] error correctors. As for the real reads, we set all the assemblers to perform scaffolding and gap closure parts to compare the quality values of the assembly results. Because ccTSA did not exploit paired-end reads, we used SSPACE [19] to scaffold contigs. We ran ccTSA and SSPACE using both datasets of preprocessed reads and reported the better assembly results. For the other assemblers compared in this paper, we used their own internal scaffolding features. We reported the NG50 values, the numbers, and the error-corrected sizes of contigs and scaffolds using the analysis tools available from the GAGE web site.

**Figure 1:**
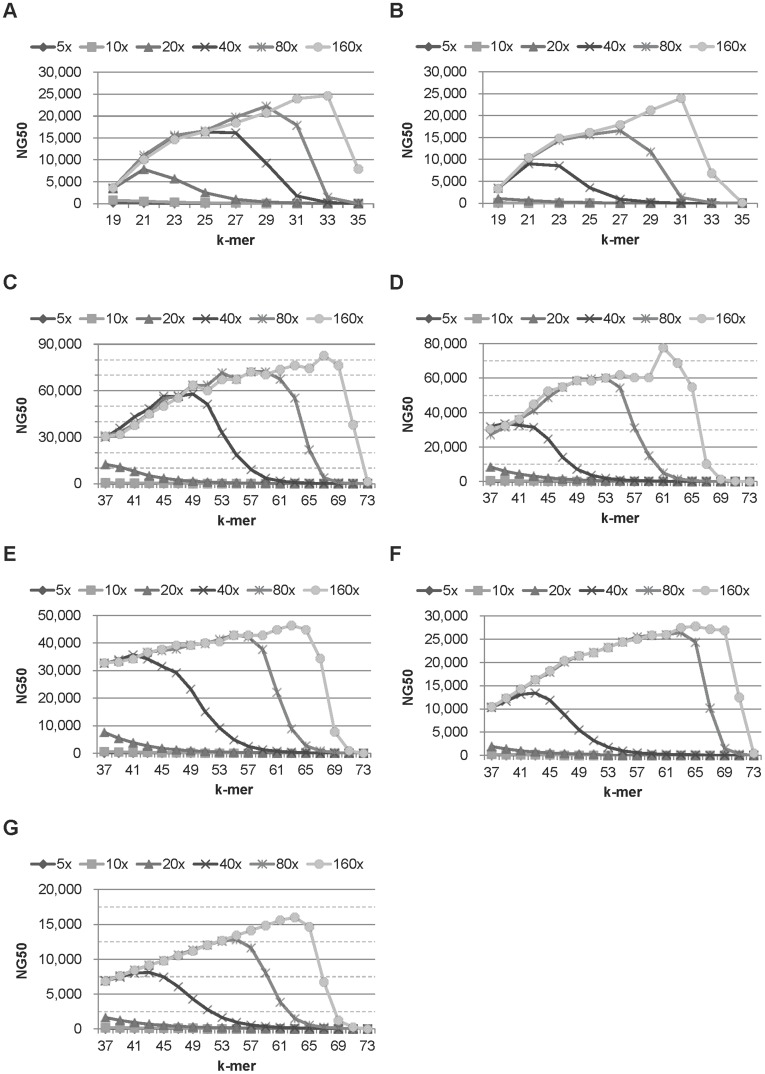
The NG50 of ccTSA on datasets from 4 organisms with different sequence coverage and k-mer values. (A) E.coli, Exact error model (Exact), and 36 bp reads (36 bp), (B) E.coli, Illumina error model (Illumina), and 36 bp, (C) E.coli, Exact, and 75 bp reads (75 bp), (D) E.coli Illumina, and 75 bp, (E) S.cerevisiae, Illumina, and 75 bp, (F) L.major, Illumina, and 75 bp, and (G) C.elegans, Illumina, and 75 bp. The k-mer values were varied from 19 to 35 on 36 bp data and from 37 to 73 on 76 bp data. In most datasets, NG50 values increased then decreased as we increased the k-mer values. The NG50 values were mostly saturated on the sequence coverage of 80x. The longer the genome size of an organism, the lower its NG50 values were.

The parallel versions of Velvet 1.2.01 [6], SOAPdenovo 1.05 [8], and ABySS 1.2.7 [7] were used for assembly. We compared the generated contigs (contiguous DNA sequences reconstructed from the assemblers) with the reference genomes using megablast [20] in NCBI BLAST+2.2.25 [21]. The parameters and configuration files used for BLAST+, Velvet, ABySS, SOAPdenovo, and ccTSA are listed in Table S4. We measured the assembler performance on a system with 4 octo-core Intel Xeon 4820 processors (total 32 computing cores) and 512GB of main memory that ran RHEL 6, gcc 4.4.4, and Open MPI 1.4.3. We used 16 hardware threads for executing the assemblers by default, and scaled the assemblers to utilize up to 32 cores. Unless mentioned otherwise, ccTSA pruned the k-mers with coverage value 1 from the k-mer coverage table before building a de Bruijn graph. We used SSPACE 1.1 [19] for scaffolding contigs generated from ccTSA.

**Figure 2:**
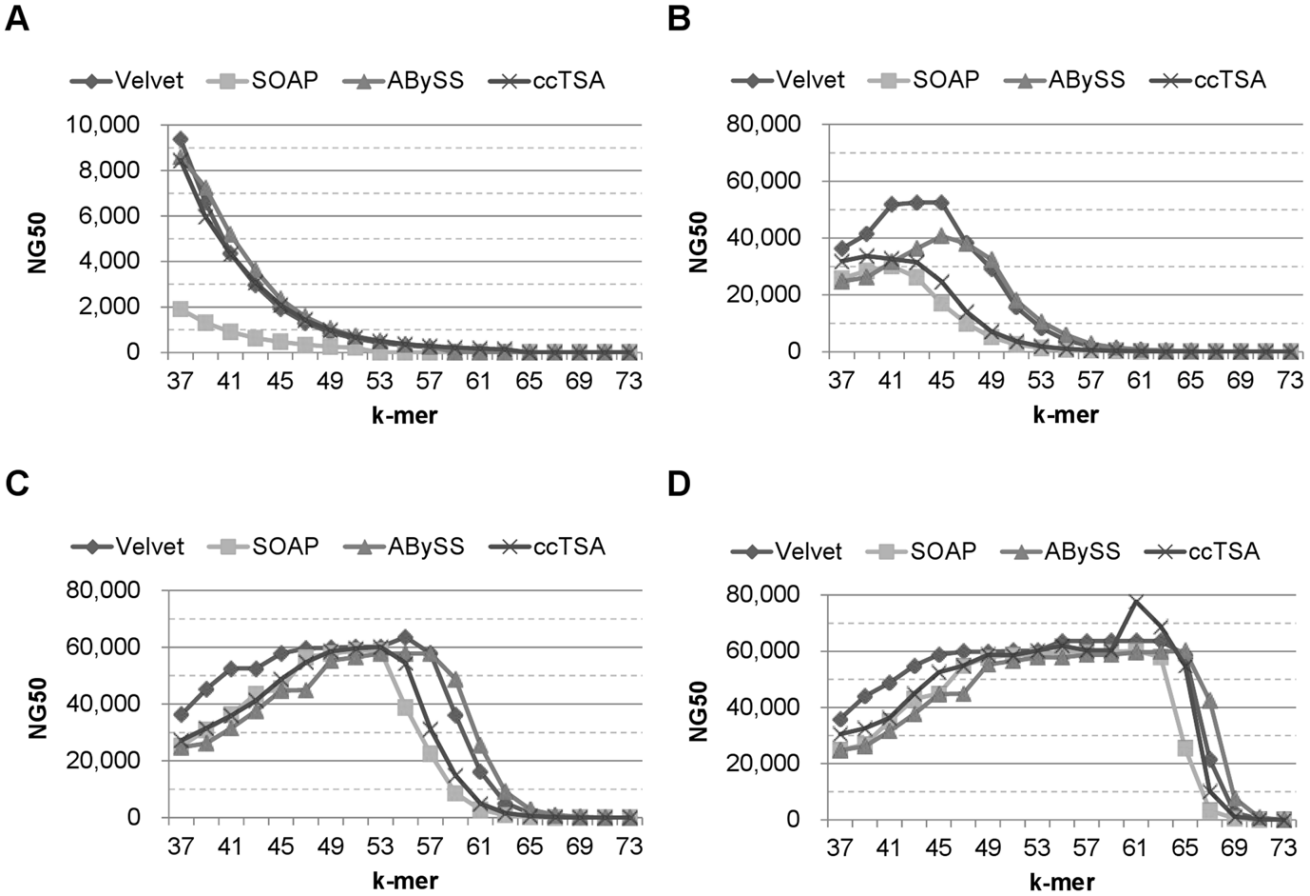
The NG50 of 4 assemblers on datasets from E.coli with different sequence coverage and k-mer values. (A) 20x, (B) 40x, (C) 80x, and (D) 160x. Illumina error model and 75 bp reads were used. Note that (C) and Figure 3(B) are the same. The NG50 values are mostly saturated on the sequence coverage of 80x for all the assemblers.

**Figure 3:**
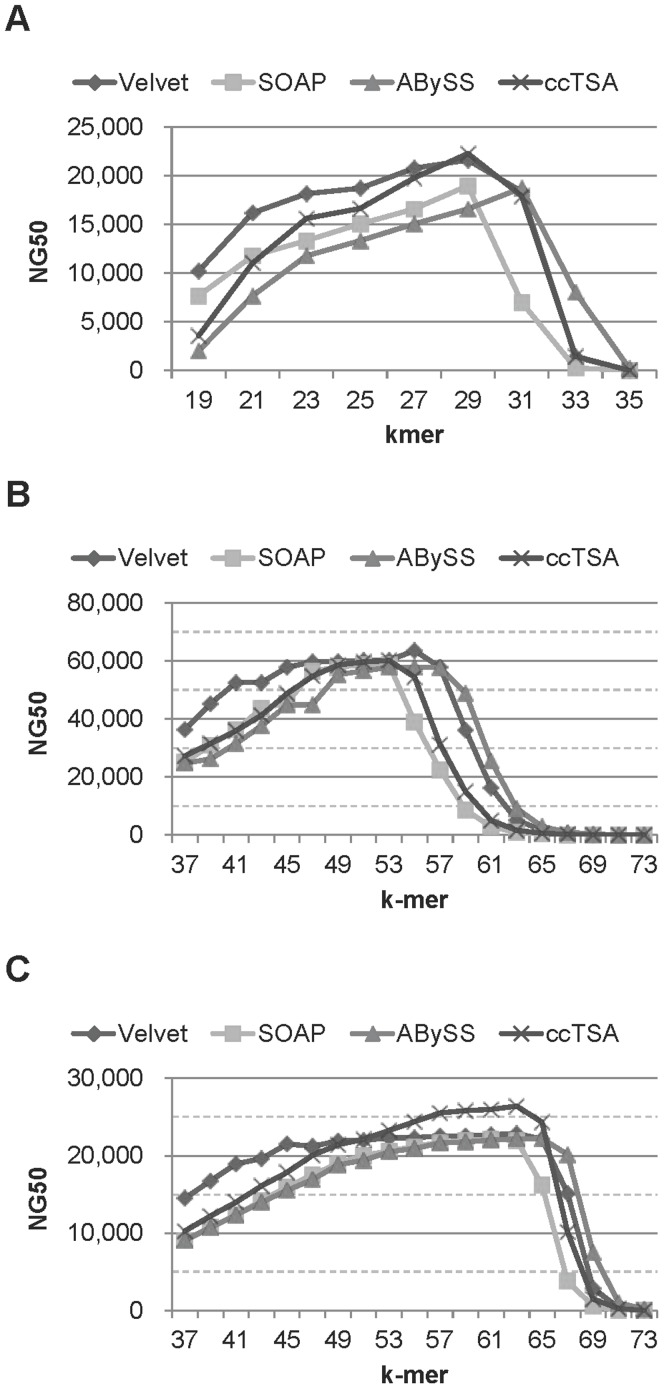
The NG50 of 4 assemblers on E.coli and L.major 80x with various k-mer values. (A) E.coli, Exact, and 36 bp, (B) E.coli, Illumina, and 75 bp, and (C) L.major, Illumina, and 75 bp. All the assemblers show similar trends on the NG50 values over various k-mer values. No single assembler produced the highest NG50 values on the entire range of k-mer values, but the NG50 values of Velvet and ccTSA were higher than others on many points.

### Evaluation

We compared the execution time, the maximum memory usage, and the quality of the generated contigs of ccTSA with other assemblers. For the experiments using the synthetic reads, we used the following quality metrics: the largest contig length (Max), N20, N50, NG50, N80, and the fraction of the genome covered by the assembled contigs, called covered genome ratio (CGR). The assembled contigs were aligned to the reference genome with NCBI BLAST+2.2.25 using megablast algorithm. Among the generated contigs, we discarded the sequences that were either lower than 98% identical to the reference or too short (shorter than 100 bases for 36 bp reads and 200 bases for 75 bp reads). We counted the bases in the genome that were mapped to the remaining contigs to compute the covered genome ratio. The NG50 value is the length of a contig when the aggregate size of the contigs that are not smaller than the contig reaches half of the reference genome length.

**Figure 4:**
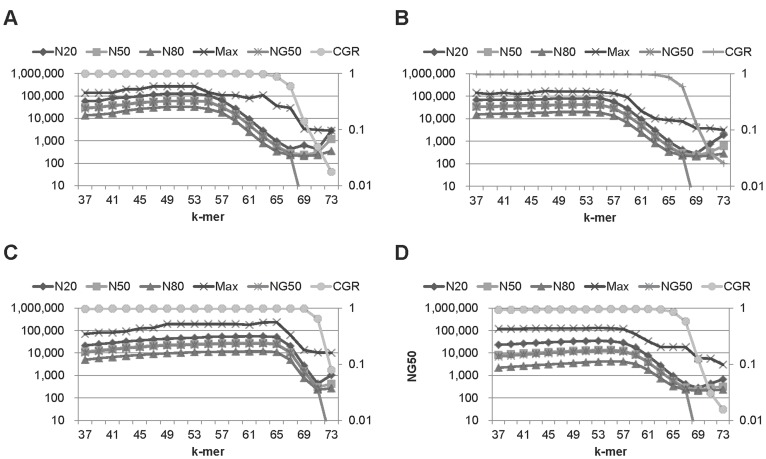
The quality values of ccTSA on 75 bp, Illumina, 80x datasets from 4 organisms with various k-mer values. (A) E.coli, (B) S.cerevisiae, (C) L.major, and (D) C.elegans. The k-mer values were varied from 19 to 35 on 36 bp data and from 37 to 73 on 76 bp data. Max stands for the largest contig length. Other quality values, such N20, N50, N80, and the largest contig length, have the trends similar to NG50.

Figure 1 shows the NG50 values ccTSA produced for datasets from 4 organisms when we varied the read length, the error model, and the sequence coverage of the synthetic reads. Figure 1A shows the NG50 of E.coli 36 bp synthetic reads without base-call errors (E.coli-Exact-36 bp) on various k-mer lengths. At a given sequence coverage, the NG50 values first increased then decreased as the k-mer length increased. As the sequence coverage increased, the NG50 values increased but were saturated starting from 80x. Also, the k-mer length giving the best NG50 value increases as the sequence coverage increases. When we introduced errors to the reads using the Illumina error model, the trends of the NG50 over the k-mer length and the sequence coverage were similar, but the NG50 values were smaller than the ones without errors (Figure 1B). When we increased the read length from 36 bp to 75 bp, the trends were unchanged, but the NG50 increased as fewer regions of a genome were aliased such that a read was mapped to multiple regions (Figure 1C and 1D). On other organisms, the trends of the NG50 were unchanged. However, the NG50 at a given sequence coverage decreased as the length of a genome increased (Figure 1E, 1F, and 1G).

**Figure 5:**
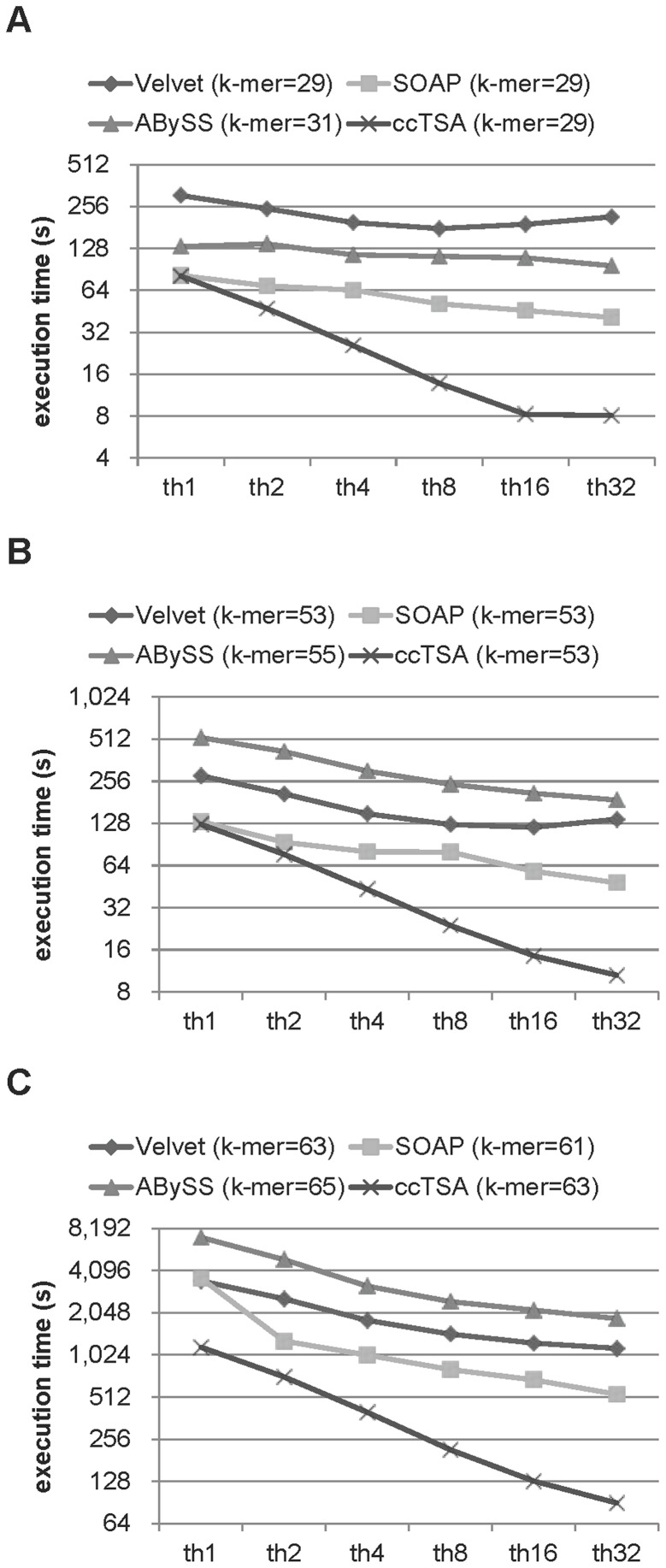
The execution time of 4 assemblers on E.coli and L.major 80x with thread numbers varied. (A) E.coli, Exact, and 36 bp, (B) E.coli, Illumina, and 75 bp, and (C) L.major, Illumina, and 75 bp. The k-mer value that produced the highest NG50 value was chosen for each assembler. As the dataset size increases, the scalability of the execution time improves. ccTSA ran faster and had better scalability in speed than the other assemblers.

**Table 2:** The contig lengths, quality, sequencing speed, and memory usage of the sequence assemblers.

	Read	Error									Time	Memory
Organism	length	Model	Assembler	k-mer	N20	N50	N80	Max	NG50	CGR	(s)	(GB)
E.coli	36x	Exact	Velvet	29	43126	23964	9880	138180	21593	0.990	164.1	1.15
			SOAP	29	41945	20161	9218	127974	18951	0.988	46.0	0.20
			ABySS	31	41813	20762	9047	127976	18720	0.988	109.9	0.50
			ccTSA	29	41950	23404	9865	129729	22251	0.982	8.3	0.71
		Illumina	Velvet	27	42081	20559	9650	120911	19112	0.989	148.4	1.06
			SOAP	27	32335	15785	7658	120913	15339	0.986	60.6	1.15
			ABySS	29	35359	17045	8015	74618	16305	0.985	144.0	1.91
			ccTSA	27	35538	17765	9474	120914	16566	0.982	14.6	2.13
	75x	Exact	Velvet	63	140955	67344	30857	326386	63602	0.996	127.5	0.74
			SOAP	59	131882	60346	30851	326380	59806	0.995	39.0	2.61
			ABySS	63	131888	60352	31019	326386	59812	0.996	140.1	1.32
			ccTSA	59	134957	73692	34655	326382	72272	0.993	8.6	0.75
		Illumina	Velvet	53	140928	67324	31713	269798	60146	0.996	120.1	2.75
			SOAP	53	123955	59642	27864	180837	58773	0.994	58.1	4.45
			ABySS	55	123957	59644	29823	269799	57834	0.994	209.5	3.23
			ccTSA	53	123953	60418	32481	269712	60146	0.998	14.5	2.74
S.cerevisiae	75x	Illumina	Velvet	55	73808	42367	19557	151220	39645	0.974	245.0	6.62
			SOAP	51	69026	37752	17390	140363	35826	0.962	169.1	9.76
			ABySS	55	69403	38353	17645	140369	36100	0.963	552.0	5.59
			ccTSA	55	82638	42169	18698	150817	39763	0.982	32.2	6.48
L.major	75x	Illumina	Velvet	63	52565	25446	10053	205626	22857	0.976	1242.6	27.02
			SOAP	61	49947	24148	9542	160354	22121	0.973	680.0	30.11
			ABySS	65	51281	24348	9710	205630	22226	0.971	2135.3	13.72
			ccTSA	63	55290	28582	11915	228670	26404	0.988	128.6	23.56
C.elegans	75x	Illumina	Velvet	55	39346	15891	4729	130754	14450	0.953	2395.0	53.49
			SOAP	53	32571	12928	3646	130752	11595	0.946	1391.0	58.40
			ABySS	57	34652	13753	3944	130760	12532	0.949	4643.0	34.63
			ccTSA	55	33498	13902	4252	125563	12817	0.962	383.1	53.97

Max stands for the largest contig length. While ccTSA produced comparable sequencing quality and superior sequencing speed, its memory usage was not much better than the other assemblers, especially compared to ABySS on large datasets.

Figure 2 shows the NG50 values from ccTSA and the other assemblers on E.coli 75 bp reads using the Illumina error model. Other assemblers showed similar trends in NG50 when the k-mer lengths and sequence coverage values were varied. The NG50 values of Velvet were higher than those of other assemblers on small sequence coverage values, but became similar when the coverage value exceeded 40x. The NG50 values on other organisms showed similar trends and were not included in this paper. Because the improvement on NG50 was marginal after the sequence coverage of 80, we used 80x reads hereafter.

**Figure 6:**
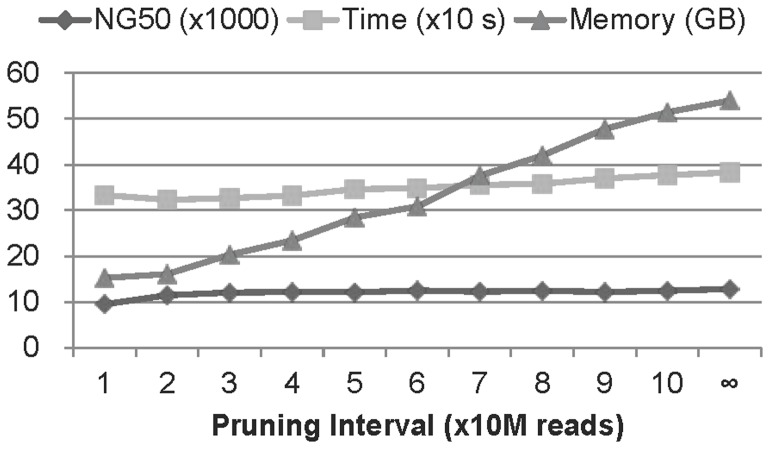
The relationship between the NG50, execution time, and memory usage of ccTSA. We used the C.elegans, Illumina, and 75 bp reads, chose the pruning interval as 10 M reads, and increased it by 10 M for subsequent configurations. Note that the dataset had about 105 M reads. As the pruning interval increases, the execution time increases slowly, the NG value improves slightly, but the memory usage grows rapidly.

We compared the NG50 values of the four assemblers on the E.coli datasets in Figure 3A and 3B. All the assemblers generated similar NG50 values on a given k-mer length. No single assembler produced the highest NG50 values on the entire range of k-mer values, but the NG50 values of Velvet and ccTSA were higher than others on many points. For the 75 bp reads with the Illumina error model, the k-mer values that provided the highest NG50 were similar: 53 for Velvet, SOAPdenovo, and ccTSA, and 55 for ABySS. The results on other organisms showed the same trends. Among them, we presented the NG50 values on L.major 80x reads with the Illumina error model in Figure 3C.

**Table 3:** The quality values of the sequence assemblers on paired-end data sets.

	Contigs				Scaffolds			
	Num	NG50 (kb)	Errors	NG50 corr (kb)	Num	NG50 (kb)	Errors	NG50 corr (kb)
S.aureus								
ABySS	302	29.2	14	24.8	246	34	1	28
SOAPdenovo	107	288.2	48	62.7	99	332	8	284
Velvet	162	48.4	28	41.5	45	762	17	126
ccTSA (k-mer = 31)	167	70.2	74	35.0	95	248.2	2	248.2
ccTSA (k-mer = 45)	103	104.8	58	42.5	51	1,565.0	6	238.6
R.sphaeroides								
ABySS	1915	5.9	55	4.2	1701	9	3	5
SOAPdenovo	204	131.7	414	14.3	166	660	3	658
Velvet	583	15.7	35	14.5	178	353	6	270
ccTSA (k-mer = 31)	350	36.2	592	9.3	144	341.8	23	149.1
ccTSA (k-mer = 29)	360	47.3	206	16.8	254	154.3	12	82.2

Two organisms, S. aureus and R.sphaeroides, were used. We used the following quality metrics, which were used for the GAGE evaluation study: the number, NG50, and corrected NG50 of the contigs and scaffolds from the assemblers as well as the number of errors. The number of misjoins and indel errors larger than or equal to 5 base pairs was counted as the errors for contigs, and the number of misjoins became the errors for scaffolds.

NG50 is not the only quality metric of the assembly results. We report other metrics, such as N20, N50, N80, the largest contig length, and the covered genome ratio (CGR), of ccTSA on 75 bp reads with the Illumina error model in Figure 4. On a given k-mer length, the aggregate contig length was the largest, followed by the longest contig length, N80, N50, NG50, and N20 on most cases as expected. The CGR of the generated contigs was higher than 95% on most k-mer lengths, which shows the usefulness of ccTSA as a sequence assembler. The CGR values ccTSA produced were also similar to those from other assemblers, as shown in Figure S1.

**Figure 7:**
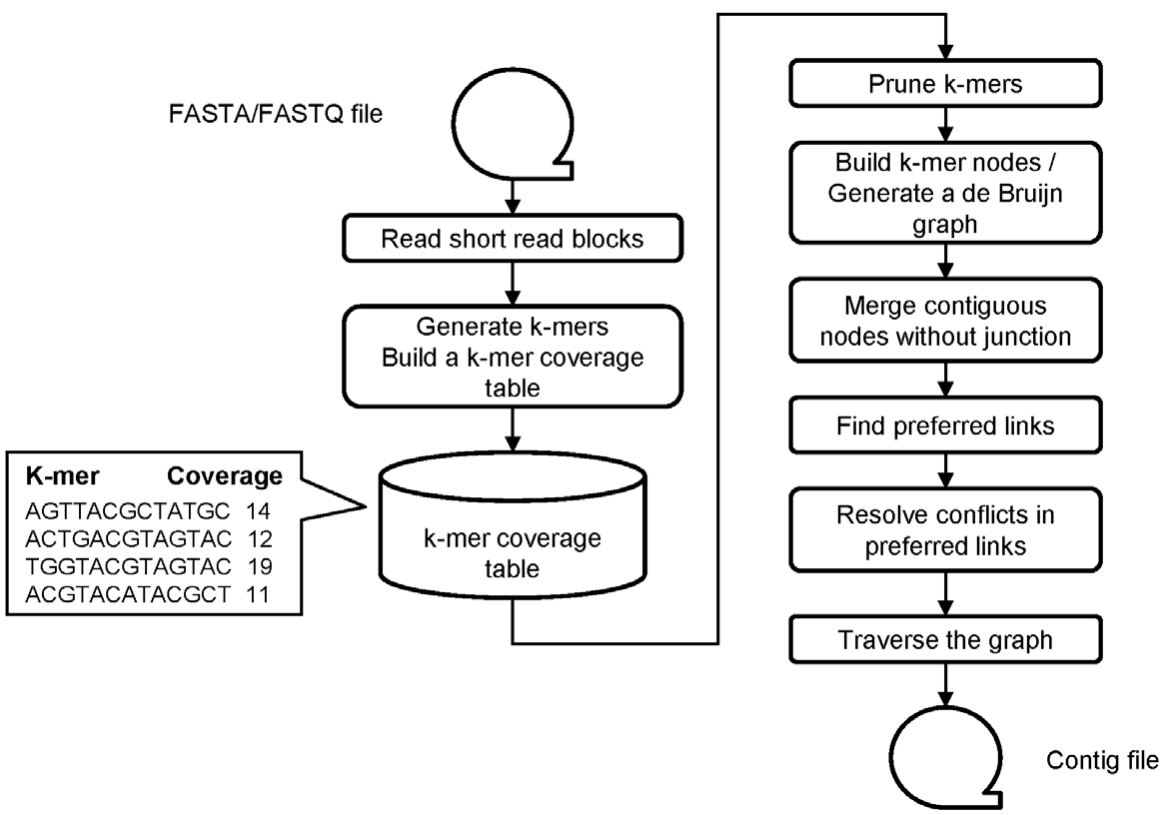
An overview of the execution flow of ccTSA. ccTSA reads short reads listed in FASTA/FASTQ files, generates k-mers from each read, and push those k-mers to a k-mer coverage table. After processing all the short reads, ccTSA optionally prunes k-mers, builds a de Bruijn graph using the remaining k-mers, merges contiguous nodes without junction, finds preferred links, resolves conflicts among preferred links, and produces contigs by traversing the graph.

Above results showed that the assembly quality, such as the NG50 and the CGR, of ccTSA was on par with or surpassed that of other sequence assemblers. We then compared the performance of the assemblers, where ccTSA provided huge advantages over the others in sequencing speed. Figure 5 shows the execution time of ccTSA, Velvet, SOAPdenovo, and ABySS, when we increased the number of utilized hardware threads from 1 to 32. On each dataset, we used the k-mer length that gave the highest NG50 value, which was also the function of the assembler. The sequencing speed was improved by utilizing multiple threads on all the assemblers and it scaled better on larger datasets, but the sequencing speed of ccTSA was substantially better than other assemblers. ccTSA was 23.1, 5.6, and 13.3 times faster than Velvet, SOAPdenovo, and ABySS, respectively, on E.coli-Exact-36 bp reads, 13.0, 4.6, and 17.9 times faster than Velvet, SOAPdenovo, and ABySS on E.coli-Illumina-75 bp reads, and 9.7, 5.3, and 16.6 times faster than Velvet, SOAPdenono, and ABySS on L.major-Illumina-75 bp reads, when 16 hardware threads were used. The sequencing speed of ccTSA also scaled better than others. When the number of threads was increased from 1 to 16, the sequencing speed of ccTSA improved 9.0 times while that of Velvet, SOAPdenovo, and ABySS improved 2.8, 5.3, and 3.3 times on L.major-Illumina-75 bp reads. Table 2 summarized the contig length, quality, sequencing speed, and memory usage of the assemblers. Even though ccTSA was substantially faster than others, it used more main memory than others except SOAPdenovo on many datasets. Because a genome could have billions of base pairs, it is important to lower the memory usage.

**Figure 8:**
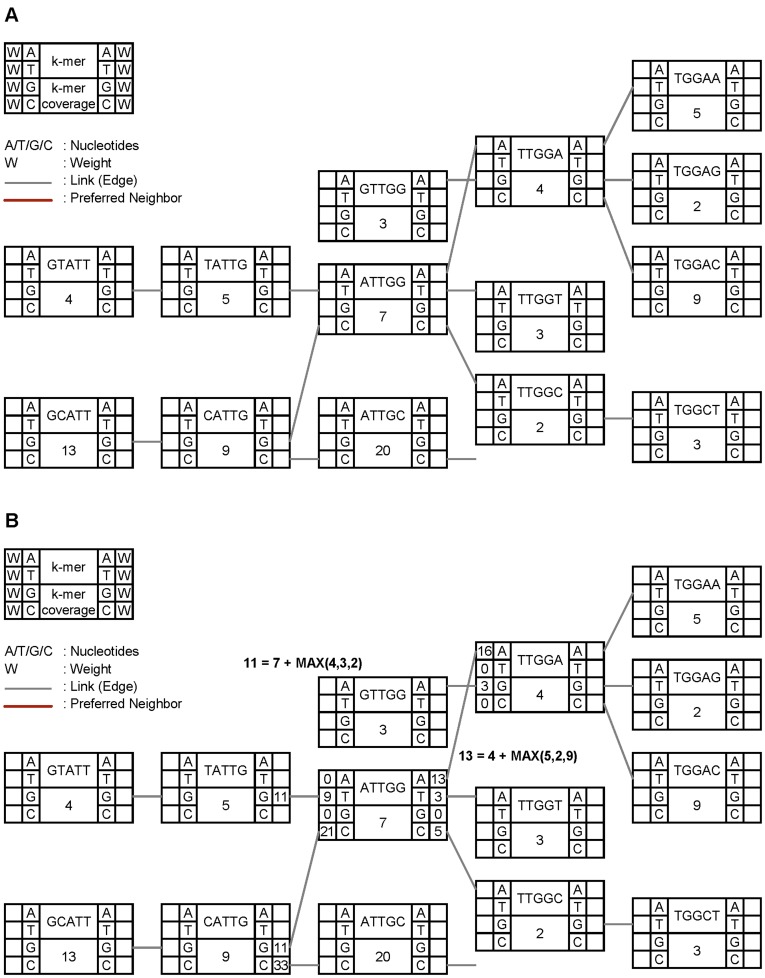
A snippet of a de Bruijn graph consisting of k-mer nodes and links. In Figure A, k-mer nodes are connected to neighbors through links. Figure B illustrates how to calculate the weight of an edge in a k-mer node. Currently, the weight of an edge on each side is computed by adding the coverage of the neighbor k-mer connected through the edge with the maximum coverage value among the k-mers connected to the neighbor k-mer on the same side.

We implemented a feature in ccTSA that trades the memory usage during execution for the quality of the generated contigs. This feature is based on the observation that the histogram of the coverage values on a k-mer coverage table reveals that a large portion of k-mers have low coverage values, mostly from base-call errors. If we prune these low coverage k-mers in the middle of building the table periodically instead of pruning them after all reads are processed, we can considerably lower the memory usage at the cost of slightly worse assembly quality due to the small possibility that the k-mers to be pruned are not from errors. If we increase the pruning frequency, low coverage k-mers are pruned more often so that ccTSA uses less memory, but the quality gets lowered as well. On the contrary, lowering pruning frequency leads to more memory usage, but better contig quality. Figure 6 showed that pruning the k-mers with coverage value 1 after processing every 50 M reads lowered the memory usage and execution time by 47.3% and 9.5%, respectively, at the cost of 5.6% degradation in NG50 compared to the default option that pruned the k-mers with coverage value 1 after finishing coverage table construction on C.elegans-Illumina-75 bp reads. Changing the pruning frequency to every 20 M reads further lowered the memory usage and execution time by 43.4% and 6.7% at the cost of additional 5.1% degradation in NG50.

**Figure 9:**
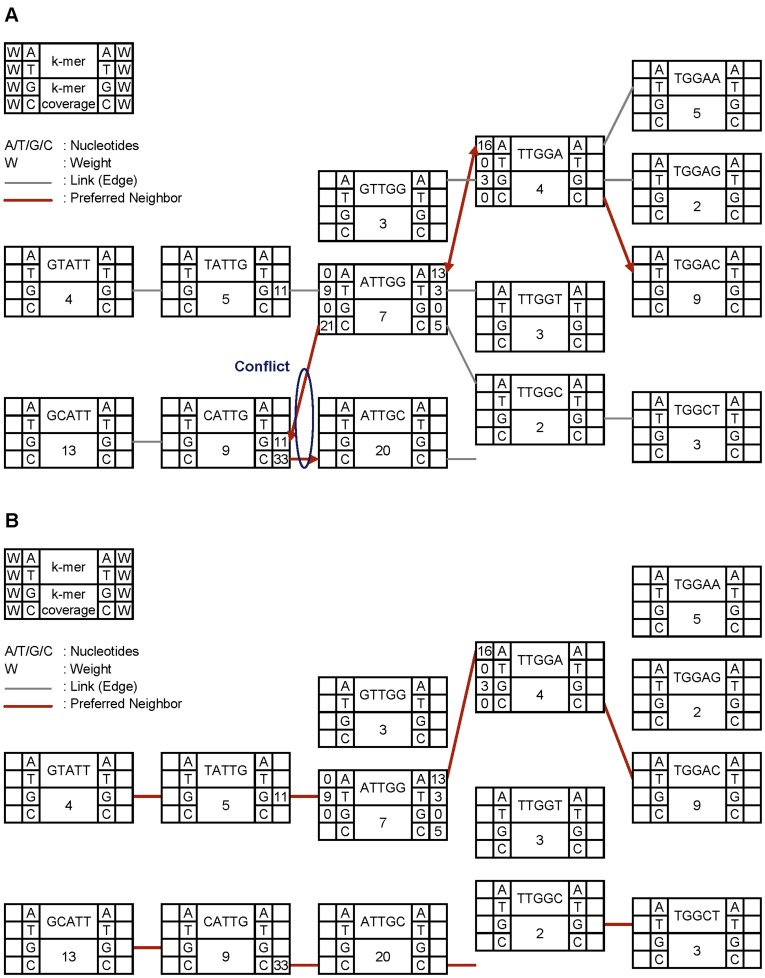
Conflict resolution between neighbor k-mer nodes. Preferred neighbors of a k-mer node, up to one on each side, are shown as thick, red-colored arrows in Figure A. There is a conflict between the ATTGG node and the CATTG node. ATTGG directs CATTG as a preferred neighbor, but CATTG directs ATTGC as a preferred neighbor. Because CATTG has a higher k-mer coverage value, the link between ATTGG and CATTG is disconnected and ATTGG finds a new preferred edge among the remaining one, which directs TATTG (Figure B).

Table 3 shows the assembly quality of ccTSA and the other assemblers on S.aureus and R.sphaeroides. ABySS, SOAPdenovo, and Velvet could exploit paired-end reads and generate scaffolds. We used SSPACE, a separate scaffolding tool, to take the output contigs from ccTSA and generate scaffolds. We configured ccTSA not to prune k-mers. We used the following quality metrics, which were used for the GAGE evaluation study: the number, NG50, and corrected NG50 of the contigs and scaffolds from the assemblers as well as the number of errors. The number of misjoins and indel errors larger than or equal to 5 base pairs was counted as the errors for contigs, and the number of misjoins became the errors for scaffolds. We broke contigs and scaffolds at each error and reported the broken ones as the corrected NG50 values. As for the results of ABySS, SOAPdenovo, and Velvet, We listed the values reported in the GAGE evaluation paper [4]. When we set the k-mer length to 31, which was the number used at the GAGE paper, the quality values of ccTSA were better than those of ABySS and comparable to those of SOAPdenovo and Velvet. By changing the k-mer length, we could find the configurations that had better quality values. For example, when we set the k-mer length to 45 base pairs, the NG50 value of S.aureus scaffolds was 1.56 million base pairs, which was much longer than those of other assemblers.

## Methods

In this section, we first provide an overview of the algorithms implemented in ccTSA. Then, we explain the techniques that exploit the characteristics of contemporary computer systems to effectively parallelize and save the memory usage of ccTSA.

### Execution Flow of ccTSA

ccTSA reads input files, each of which is composed of the short fragments (reads) of an original DNA sequence, and generates an output file that contains the result of sequence assembly. Sequencing machines [3] occasionally make mistakes in reading base-pairs, which are called base-calling errors, and some k-mers are mapped to the multiple regions of the original sequence, which are called repeats. As a result, it is not always possible for a sequence assembler to perfectly reconstruct the original sequence. So the output file of ccTSA typically consists of multiple DNA sequences called contigs and none of the contigs might be mapped to some regions of the original sequence. Currently, ccTSA can read FASTA and FASTQ files and writes the generated contigs to a FASTA file.

Figure 7 illustrates an overview of the execution flow of ccTSA, which consists of multiple phases. First, it reads the series of short reads and extracts k-mers from each read. Because a k-mer consists of k nucleotides, a read that has fewer than k nucleotides is discarded. ccTSA also discards k-mers that have ambiguous or unidentified nucleotides. It checks a dictionary called a k-mer coverage table, which has a k-mer as a key and its coverage as a value, to see if the extracted k-mer exists in the table. If so, its coverage value is incremented by one. If not, the k-mer is added to the table with the coverage value 1. Note that k-mer coverage is different from the sequence coverage of the original DNA sequence. The former is the number of a k-mer instance from the sequenced reads, while the latter stands for how many times a nucleotide in the original sequence appears at the reads.

After all the reads are processed, ccTSA optionally prunes k-mers with too low or high coverage values. Assuming that the original sequence consists of g nucleotides, the k-mer coverage table would have (g–k+1) entries if the sequence has no repeats and the reads have no base-calling errors. If a k-mer generated from a sequenced read contains one or more base-calling errors, the k-mer typically has very low coverage because it is unlikely that the original DNA sequence includes the k-mer. When the base-calling error rate of the reads is high, the k-mer coverage table has much more than (g–m+1) entries. If the coverage table has more entries, more memory space is required and it takes more time to access and update the table. Assuming that the coverage of the original DNA sequence is sufficiently high, most of low coverage k-mers are due to base-calling errors and most of high coverage k-mers are from the original sequence. As a result, pruning these low coverage k-mers can be useful for removing the base-calling errors, saving memory usage and improving sequencing speed. However, because the coverage of the original sequence is not uniform over all the nucleotides, some of the low coverage k-mers could be from the original sequence hence pruned incorrectly. This lowers the average length of the generated contigs, but it would be possible to restore them during phases after assembly, such as the scaffolding phase, which will be further discussed later in this section. k-mers with very high coverage are typically from repeats, so we can optionally mark them as repeats and exclude them hereafter.

Remaining k-mers become k-mer nodes, among which the nodes that share k-1 nucleotides are linked together through edges building a de Bruijn graph. Because there are 4 types (Adenine, Thymine, Guanine, and Cytosine) of nucleotides in DNA, a k-mer node has up to 8 neighbors, 4 to the left side that share the first k-1 nucleotides and 4 to the right side that share the last k-1 ones. A node that has multiple neighbors to either side is called a junction node. After linking, the k-mers that are connected without any junction are merged, forming a contig node.

Then, for each side of a node, the weights of the edges are computed and the neighbor with the highest weight is called a preferred neighbor. The weight of an edge represents the likelihood of the neighbor, which is highly correlated to the coverage of the neighbor nodes. As of now, the weight of an edge on each side is computed by adding the coverage of the neighbor k-mer connected through the edge with the maximum coverage value among the k-mers connected to the neighbor k-mer on the same side (Figure 8). This gives a priority to the neighbor node with higher k-mer coverage, at the same time prefers a longer path and enables ccTSA not to miss a strong or more likely path that is connected through a low coverage k-mer. ccTSA is designed to easily implement other ways to calculate weights.

After finding preferred neighbors, we check each junction node JN1 whether its preferred neighbor JN2 also points JN1 back as a preferred neighbor. If not, we call that there is a conflict between JN1 and JN2, which is resolved as follows: if the coverage of JN1 is higher, we enforce JN2 to point JN1 as a preferred neighbor; if the coverage of JN2 is higher, we disconnect the edge between JN1 and JN2, find the preferred neighbor among the remaining edges, and repeat the above steps until there is still a conflict. Our conflict resolution algorithm (Figure 9) is simpler than those of other assemblers such as tip removal and tour bus algorithms [6] in Velvet, ABySS, and SOAPdenovo. It is a future work to refine the conflict resolution algorithm. After all conflicts are resolved, finally, contigs are generated by traversing the nodes connected through preferred neighbors. Unlike other assemblers, ccTSA does not exploit paired-end reads to orient and align multiple contigs into a single super-contig or scaffold. ccTSA can leverage a separate tool, such as SSPACE [19], or the part of other assemblers to perform this scaffolding and finishing phase.

### Optimizations

Continuous improvement in semiconductor process technology enables a single chip to integrate billions of transistors and a rack server to have dozens of computing cores and terabytes of shared memory [11]. We assume that the entire working set of ccTSA fits in a shared memory space. This simplifies programming and provides better performance than the systems that distribute k-mer entries across a cluster of computers connected over a network such as InfiniBand or Ethernet [11]. Any computing core can access any k-mer entry through low latency (tens of nanoseconds) memory loads and stores in a shared memory system, while the k-mer information must be encapsulated by request and reply packets and transferred over a high latency (a few microseconds or more) network. Because the size of a k-mer entry is rather small, the overhead of packing and unpacking the entry is relatively high, further reducing program speed.

We apply several optimization techniques to ccTSA. To reduce execution time, we parallelize the phases where we construct the k-mer coverage table, populate and link k-mer nodes, and merge consecutive k-mers without junction, which take 99% of the single threaded execution of ccTSA on average over the Illumina-75 bp-80x datasets from 4 organism explained in the Results and Discussion section. When each phase is started, we first divide workload into many small chunks, each having the same size, and spawn multiple worker threads. Each worker repeats the process of receiving a chunk, processing it, and asking for another chunk that is not processed yet until all the chunks are processed. Because time for a thread to access data heavily depends on the address, the internal status of a complicated memory system within a processor, and interaction with concurrent accesses from other threads, time to process a chunk is not the same either [12]. As a result, statically dividing the workload into the worker threads suffers from the load balancing problem, while dynamically assigning chunks to idle threads leads to better performance [12]. As more worker threads are used, the performance advantage of the dynamic load balancing method becomes even higher. At the k-mer coverage construction phase, the workload is the sequences of short reads. We compose the k-mer coverage table of thousands of hash maps and use two different hash functions to identify a hash map and an entry in the hash map. Each hash map is protected by a mutex to prevent a simultaneous access to a hash map by multiple worker threads from destroying the data structure. Because there are much more hash maps than the worker threads and hash-map update is a simple operation, the worker threads rarely access the same hash map at the same time. As a result, the mutex operations do not incur significant performance overheads. Still, it is possible to further alleviate the overheads. Because a mutex is designed to protect a block of memory, not just a single word, it is heavier than an atomic CPU operation, which reads, modifies, and writes a word atomically. When the length of a k-mer is shorter than 32 base pairs, it can be represented as a single 64-bit word. Jellyfish [22] exploited this to replace the mutex operations into atomic memory operations, such as compare-and-swaps, in building concurrent hash maps and updating k-mer coverage values, and achieved a higher k-mer coverage construction performance for short k-mers. At the k-mer node populating, linking, and merging phases, each hash map becomes a chunk. Mutexes are not needed for these phases because no data is updated concurrently by the multiple threads.

To save memory usage, ccTSA compares a k-mer with its reverse complement and only stores the value which is earlier in the lexicographical order. It utilizes bit fields extensively and has different data structures for the k-mer nodes with and without junctions. It includes a custom memory allocator [14], which provides multiple allocation classes. Each class is implemented as a chain of memory blocks. When the custom allocator is used to allocate an object, the object is categorized into a class and stored at the last block of the class. If the block does not have enough free space, the default memory allocator in C++ is used to allocate a block to the class. It cannot deallocate a single object, but can quickly deallocate all the objects of a certain class simply by freeing the blocks of the class. ccTSA utilizes this custom allocator in pruning low coverage k-mers by having separate tables for low coverage and high coverage k-mers, assigning the low coverage k-mer objects and the k-mer coverage table for them to a same class, and deallocating the class. The remaining k-mer coverage table has fewer entries than the table without pruning, which has fast access time. So pruning also helps reducing execution time. We can even prune low coverage k-mers in the middle of building the k-mer coverage table, not just at the end, which provides an interesting tradeoff between the memory footprint and assembly quality, which is evaluated in the Results and Discussion section.

### Availability and Future Directions

ccTSA is written in C++ and can be run on Unix-like systems. Source code is freely available from http://code.google.com/p/cctsa/. ccTSA can be extended to multiple directions. First, alternative data structures and algorithms can be explored in search of better sequencing speed and lower memory usage. Second, ccTSA does not target current General-Purpose computing on Graphics Processing Units (GPGPUs) [11] because they do not provide enough memory capacity. However, it would be interesting to see if ccTSA can take advantage of their high computation power and memory bandwidth once future GPGPUs or many integrated core systems address the memory capacity issue. Third, ccTSA can be integrated with other scaffolding tools or extended to exploit paired-end reads to further orient and align the contigs.

## Supporting Information

Figure S1 The covered genome ratio of assemblers on E.coli and L.major 80x with various k-mer values. (A) E.coli, Exact, and 36 bp, (B) E.coli, Illumina, and 75 bp, and (C) L.major, Illumina, and 75 bp. The covered genome ratio (CGR) was more than 95% over most k-mer values regardless of the assemblers used.
(TIFF)

Table S1 Datasets used for generating synthetic reads. The chromosome data of Caenorhabditis elegans (C.elegans), Escherichia coli str. K-12 substr. DH10B (E.coli), Leishmania major strain Friedlin (L.major), and Saccharomyces cerevisiae S288c (S. cerevisiae) were downloaded from NCBI Genome Sequence. Detailed information of L.major is listed in Table S2.
(TIFF)

Table S2 Datasets used for generating synthetic reads. The chromosome data of Leishmania major strain Friedlin (L.major) were downloaded from NCBI Genome Sequence. Detailed information of C.elegans, E.coli, and S.cerevisiae is listed in Table S1.
(TIFF)

Table S3 MetaSim options used to generate synthetic reads.
(TIFF)

Table S4 Parameters and configuration files used for BLAST+, Velvet, ABySS, SOAPdenovo, ccTSA, and SSPACE.
(TIFF)

## References

[1] Miller JR, Koren S, Sutton G (2010). Assembly algorithms for next-generation sequencing data. Genomics..

[2] Butler J, MacCallum I, Kleber M, Shlyakhter IA, Belmonte MK (2008). ALLPATHS: de novo assembly of whole-genome shotgun microreads. Genome Research..

[3] Elaine RM (2008). Next-Generation DNA Sequencing Methods. Annu. Rev. Genom. Human Genet. 9: 387–402..

[4] Salzberg SL, Phillippy AM, Zimin A, Puiu D, Magoc T (2011). GAGE: A critical evaluation of genome assemblies and assembly algorithms. Genome Research..

[5] Smith TF (1980). Identification of common molecular subsequences. J. Mol. Bio..

[6] Daniel RZ, Birney E (2008). Velvet: Algorithms for de novo short read assembly using de Bruijn graphs. Genome Research..

[7] Simpson JT, Wong K, Jackman SD, Schein JE, Jones SJ (2009). ABySS: A parallel assembler for short read sequence data. Genome Research. 19(6): 1117–1123..

[8] Li R, Zhu H, Ruan J, Qian W, Fang X (2009). De novo assembly of human genomes with massively parallel short read sequencing. Genome Research..

[9] Jackson BG, Regennitter M, Yang X, Schnable PS, Aluru S (2010). Parallel de novo assembly of large genomes from high-throughput short reads. 2010 IEEE International Symposium on Parallel & Distributed Processing (IPDPS)..

[10] Wenyu Z, Jiajia C, Yang Y, Yifei T, Jing S (2011). A Practical Comparison of De Novo Genome Assembly Software Tools for Next-Generation Sequencing Technologies. PLoS ONE. 6(3): e17915..

[11] Hennessy JL, Patterson DA (2011). Computer Architecture, 5th Edition: A Quantitative Approach. Morgan Kaufmann.. 708 p.

[12] Culler D, Singh JP, Gupta A (1998). Parallel Computer Architecture: A Hardware/Software Approach. Morgan Kaufmann.. 1056 p.

[13] Pevzner PA, Tang H, Waterman MS (2001). An Eulerian path approach to DNA fragment assembly. Proc. Natl. Acad. Sci..

[14] Berger ED, Zorn BG, McKinley KS (2002). Reconsidering custom memory allocation. ACM SIGPLAN conference on Object-oriented programming, systems, languages, and applications..

[15] Richter DC, Ott F, Auch AF, Schmid R, Huson DH (2008). MetaSim-A Sequencing Simulator for Genomics and Metagenomics. PLoS ONE..

[16] Plantagora Template website.. http://www.plantagora.org/tools_downloads/read_simulation.html.

[17] Kelley DR, Schatz MC, Salzberg SL (2010). Quake: Quality-aware detection and correction of sequencing errors. Genome Biol 11: R116..

[18] Gnerre S, Maccallum I, Przybylski D, Ribeiro FJ, Burton JN, et al. 2011. High-quality draft assemblies of mammalian genomes from massively parallel sequence data.. Proc Natl Acad Sci.

[19] Marteb B, Christiaan VH, Hans JJ, Derek B, Walter P (2010). Scaffolding pre-assembled contigs using SSPACE. Bioinformatics. 27(4): 578–579..

[20] Zhang Z, Schwartz S, Wagner L, Miller W (2000). A greedy algorithm for aligning DNA sequences. J Comput Biol..

[21] Stephen FA, Warren G, Webb M, Eugene WM, David JL (1990). Basic local alignment search tool. J. Mol. Bio. 215(3): 403–410..

[22] Marçais G, Kingsford C (2011). A fast, lock-free approach for efficient parallel counting of occurrences of k-mers. Bioinformatics. 27 (6): 764–770..

