# Correction: ccTSA: A Coverage-Centric Threaded Sequence Assembler

**DOI:** 10.1371/annotation/6d56179d-39e8-4ee8-bf3e-ceab773bab20

**Published:** 2012-09-19

**Authors:** Jung Ho Ahn

There were errors in the Funding section. The correct funding information is as follows: This work was supported by the Seoul National University Brain Fusion Program Research Grant and Basic Science Research Program through the National Research Foundation of Korea (NRF) funded by the Ministry of Education, Science and Technology (2012R1A1B4003447). The funders had no role in study design, data collection and analysis, decision to publish, or preparation of the manuscript.

