# Supplementary figures and images for: ccTSA: A Coverage-Centric Threaded Sequence Assembler
Source: PLoS One. 2012 Jun 19;7(6):e39232. doi: 10.1371/journal.pone.0039232 (PMC3378524; doi:10.1371/journal.pone.0039232)

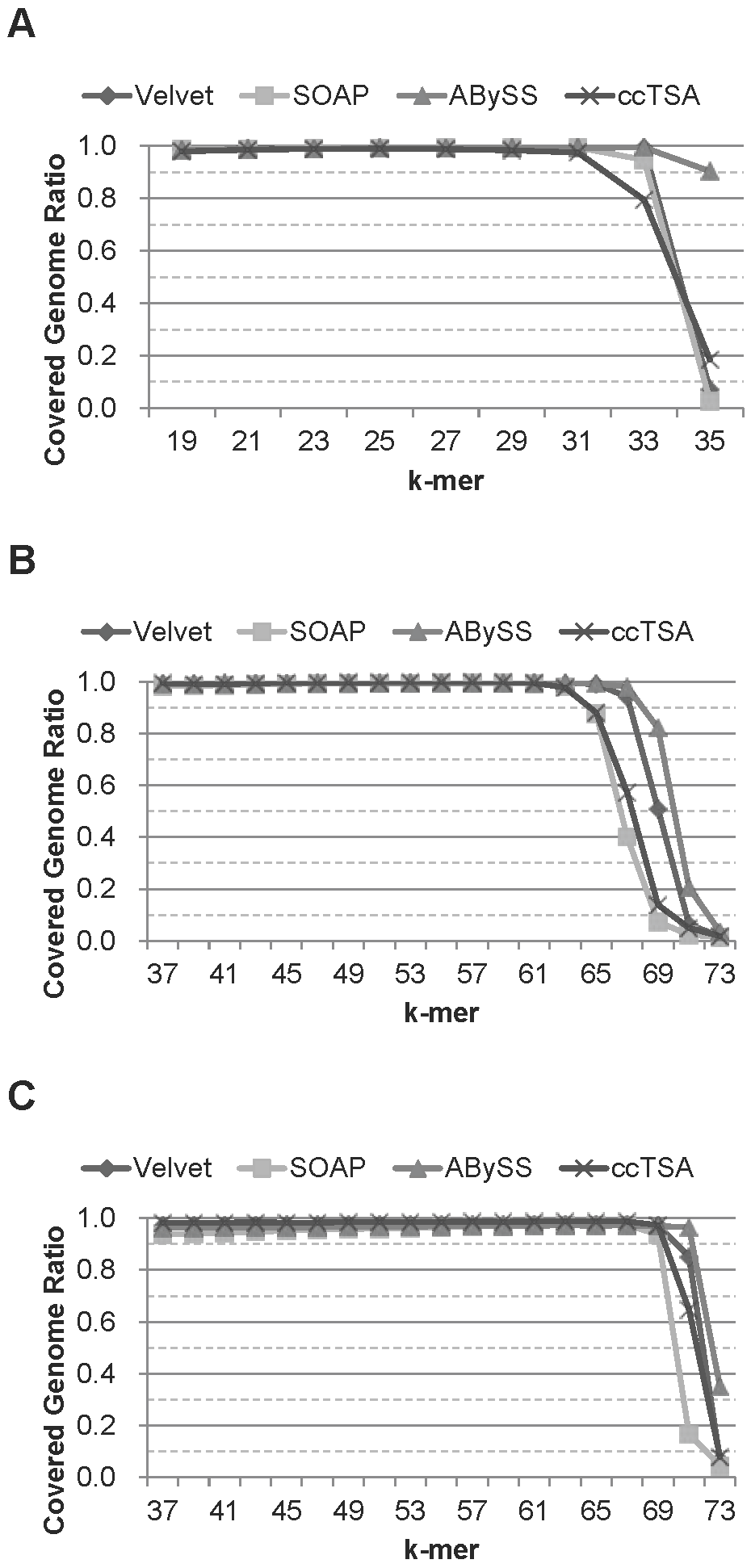

Supplement: Figure S1 — The covered genome ratio of assemblers on E.coli and L.major 80x with various k-mer values. (A) E.coli, Exact, and 36 bp, (B) E.coli, Illumina, and 75 bp, and (C) L.major, Illumina, and 75 bp. The covered genome ratio (CGR) was more than 95% over most k-mer values regardless of the assemblers used. (TIFF) [file pone.0039232.s001.tiff]

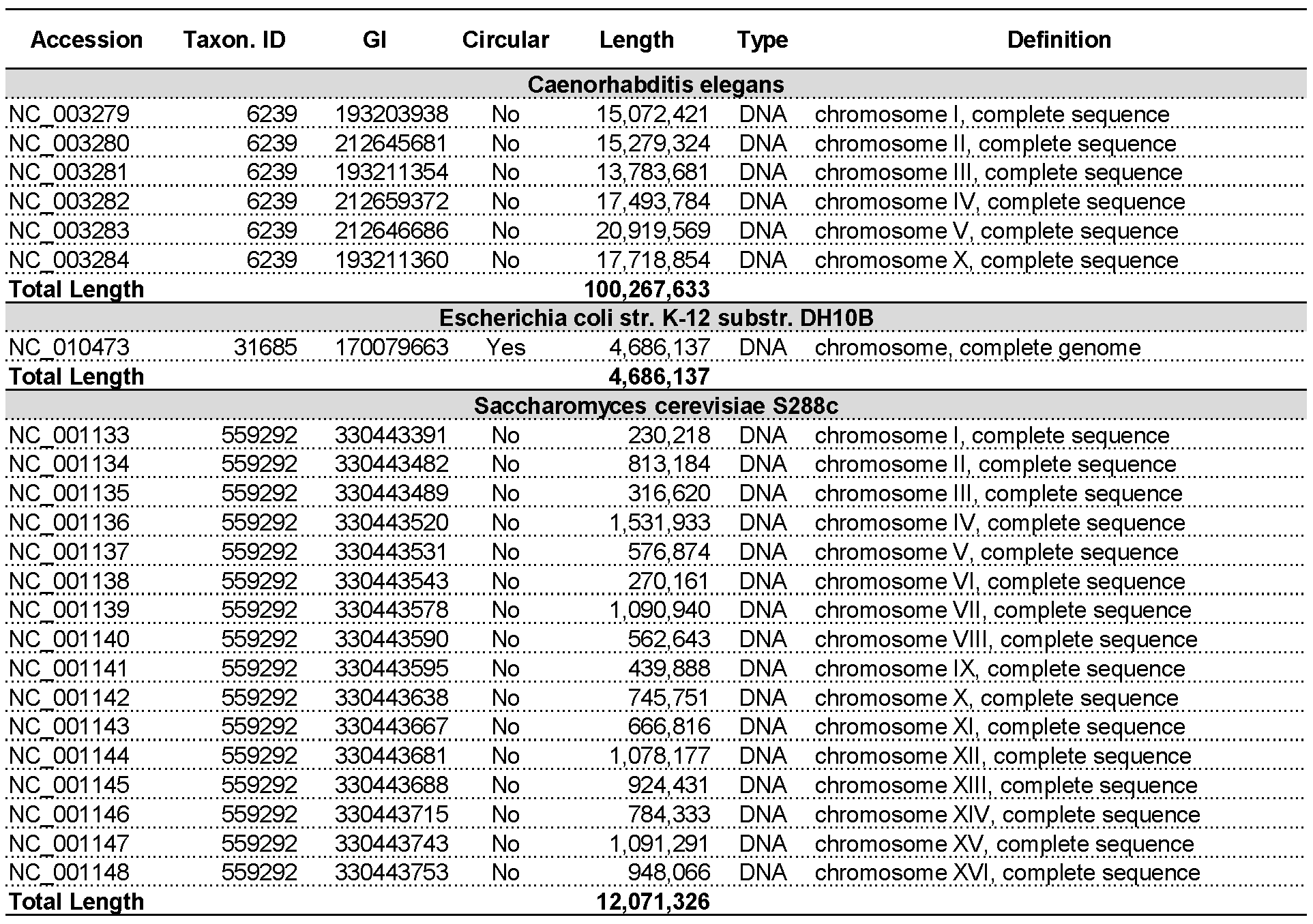

Supplement: Table S1 — Datasets used for generating synthetic reads. The chromosome data of Caenorhabditis elegans (C.elegans), Escherichia coli str. K-12 substr. DH10B (E.coli), Leishmania major strain Friedlin (L.major), and Saccharomyces cerevisiae S288c (S. cerevisiae) were downloaded from NCBI Genome Sequence. Detailed information of L.major is listed in Table S2. (TIFF) [file pone.0039232.s002.tiff]

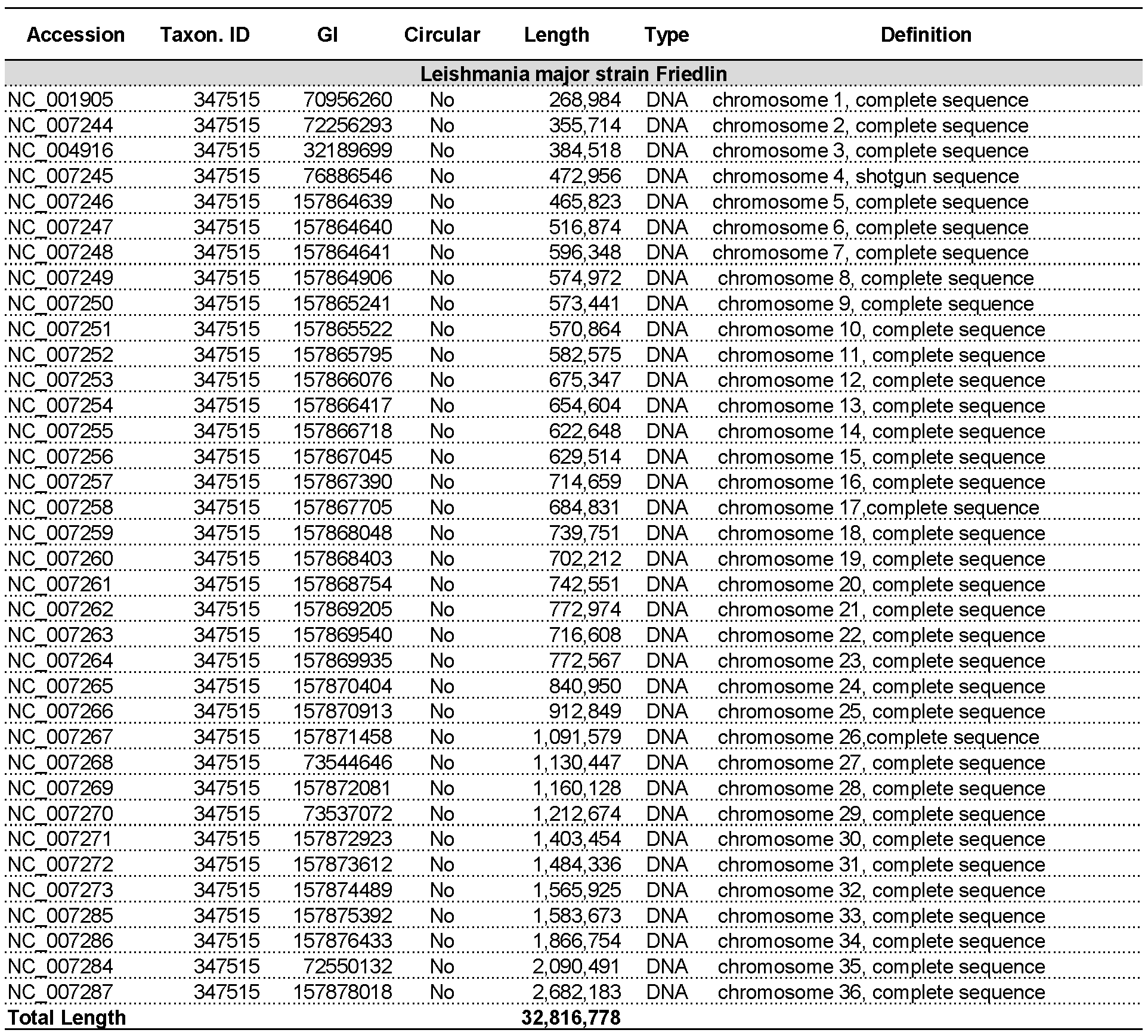

Supplement: Table S2 — Datasets used for generating synthetic reads. The chromosome data of Leishmania major strain Friedlin (L.major) were downloaded from NCBI Genome Sequence. Detailed information of C.elegans, E.coli, and S.cerevisiae is listed in Table S1. (TIFF) [file pone.0039232.s003.tiff]

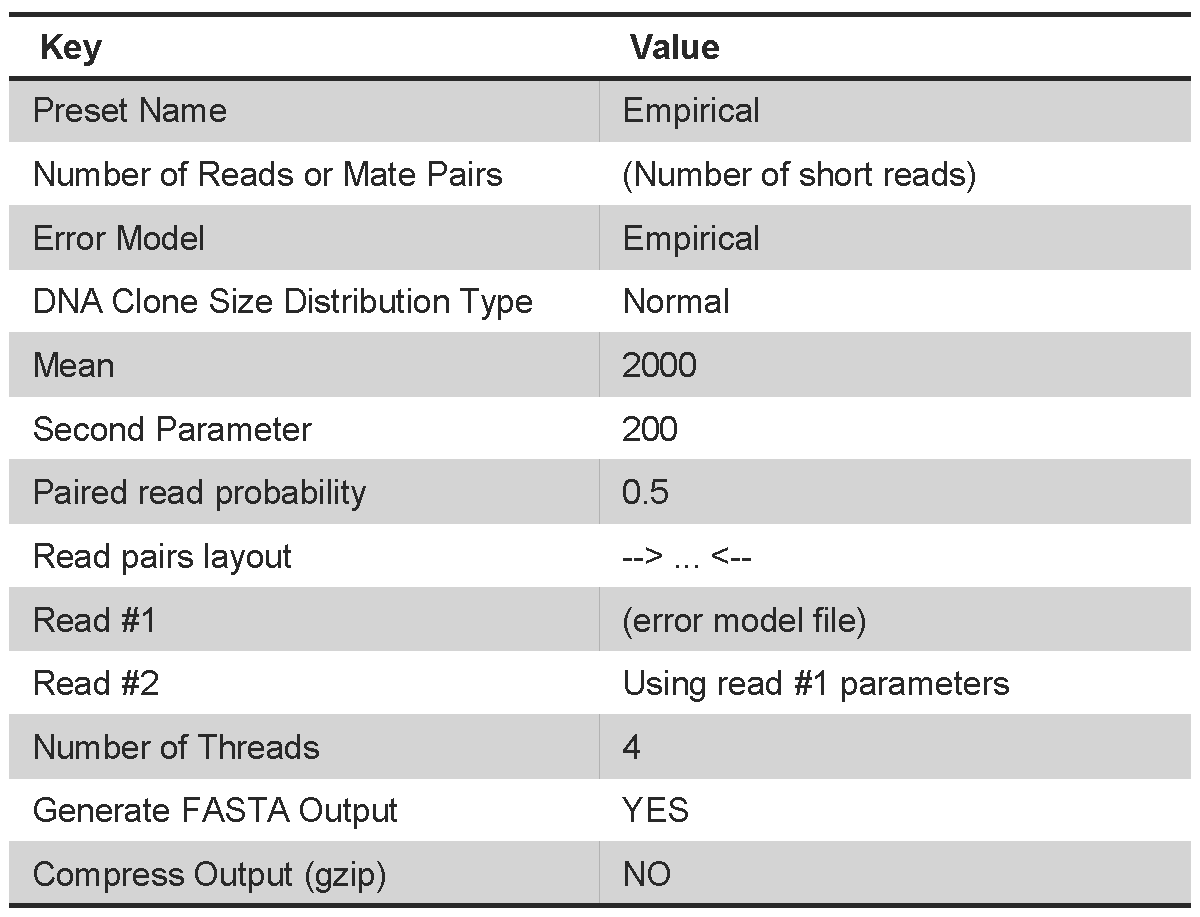

Supplement: Table S3 — MetaSim options used to generate synthetic reads. (TIFF) [file pone.0039232.s004.tiff]

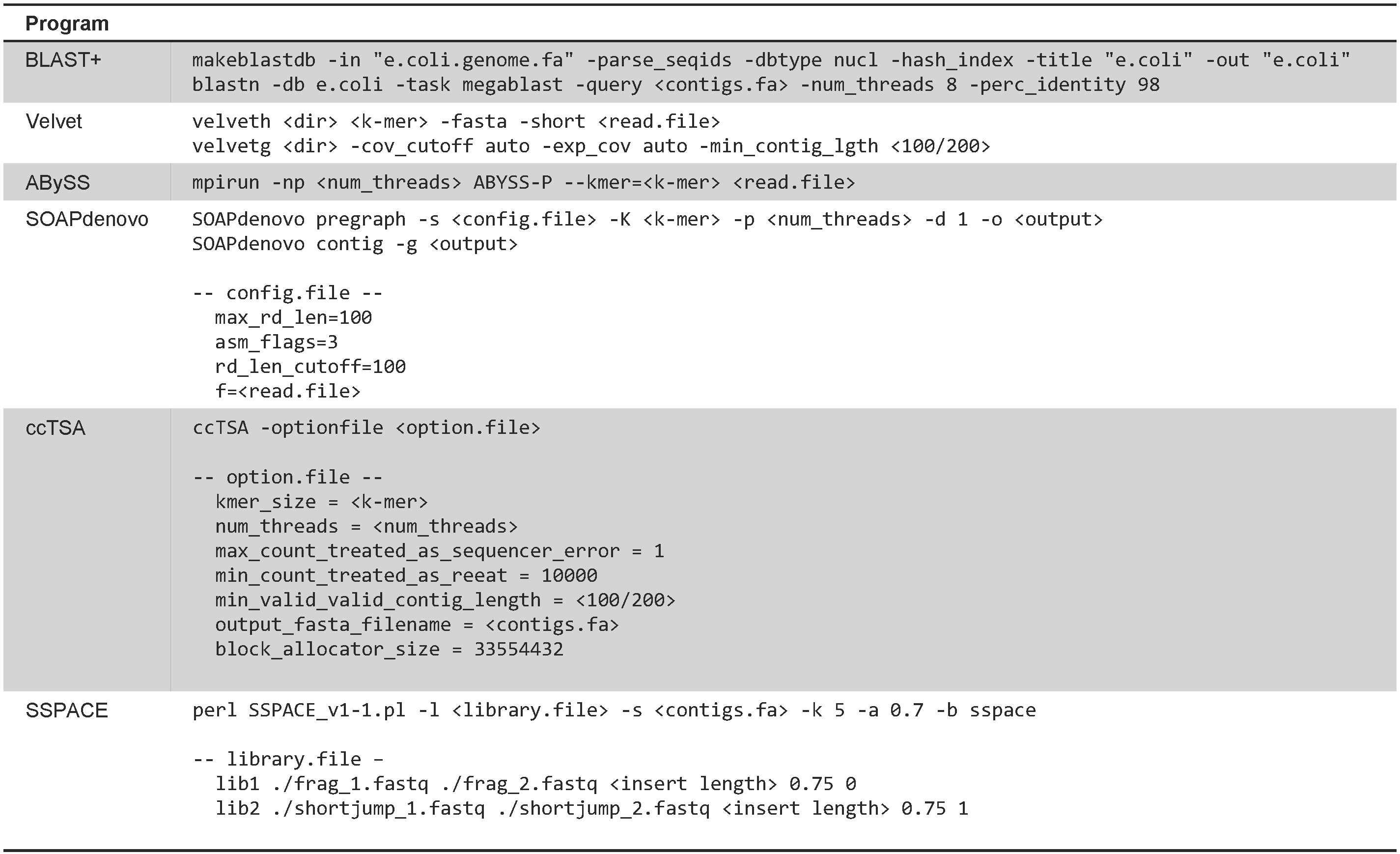

Supplement: Table S4 — Parameters and configuration files used for BLAST+, Velvet, ABySS, SOAPdenovo, ccTSA, and SSPACE. (TIFF) [file pone.0039232.s005.tiff]
